# In Commemoration of Dr. Farrokh Modabber: An Iranian Pioneer of Cellular Immunology, and Leishmaniases Vaccine Research in Iran and the World

**DOI:** 10.34172/aim.28959

**Published:** 2024-09-01

**Authors:** Fariborz Bahrami, Ehsan Mostafavi

**Affiliations:** ^1^Department of Immunology, Pasteur Institute of Iran, Tehran, Iran; ^2^Research Centre for Emerging and Reemerging Infectious Diseases, Pasteur Institute of Iran, Tehran, Iran; ^3^Department of Epidemiology and Biostatistics, Pasteur Institute of Iran, Tehran, Iran

**Keywords:** History of immunology, Iran, Leishmaniasis, Pasteur Institute of Iran

## Abstract

Born in 1940 in Rasht, Iran, Dr. Farrokh Modabber earned his B.A. in Bacteriology and Ph.D. in Microbiology from University of California, Los Angeles (UCLA). He joined the Harvard Medical School as a fellow before transitioning to a faculty role at Harvard School of Public Health (HSPH) and played a pivotal role in advancing Cellular Immunology. In the early 1970s, he returned to Iran as an Associate Professor at Pahlavi University in Shiraz. Subsequently, he rejoined HSPH before embarking on a tenure at Tehran University. As the head of the Pathobiology Department at Tehran University School of Public Health, he initiated the Tehran/Harvard joint M.Sc. program in Immunology, which played a crucial role in shaping the careers of numerous Iranian immunologists over the following decades. Dr. Modabber went on to hold esteemed positions such as Director General of the Pasteur Institute of Iran, visiting immunology lecturer at various universities, Coordinator of Research Capability Strengthening of WHO’s Special Program for Research and Training in Tropical Diseases (TDR), Director of the Infectious Disease Research Institute (IDRI), and Senior Advisor of Drugs for Neglected Diseases initiative (DNDi), to name a few. This article highlights Dr. Modabber’s impactful career, focusing on his efforts to combat global leishmaniasis.

## Early Life and Education

**Figure 1:**
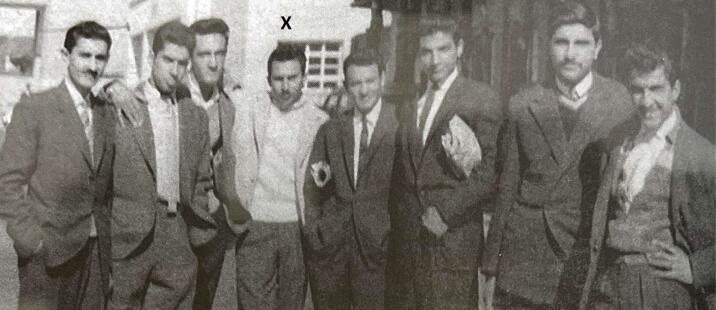
Farrokh Modabber (marked by X) and His Fellow Alborz High School Graduates (1957)^1^

 Farrokh Modabber was born on February 26, 1940, in Rasht, Iran. He moved to Tehran after the first grade of Elementary School with his family. Farrokh grew up and studied in Tehran until he graduated from Alborz High School, one of the most prestigious educational institutions in the country (Figure 1).

**Figure 2:**
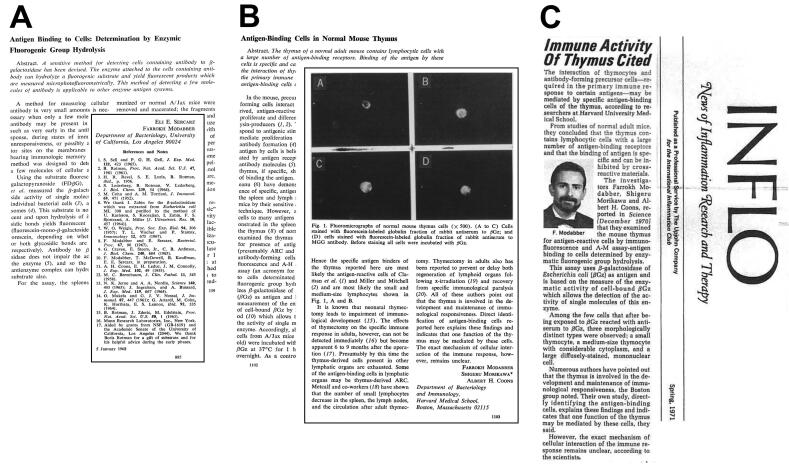
Farrokh Modabber’s Early Publications: (**A**) Publication in Science (1968) by Sercarz and Modabber^2^ (**B**) Publication in Science (1970) by Modabber et al.^3^ **(C)** INFLO News of Inflammation Research and Therapy reported on Farrokh Modabber’s research on the immune activity of thymus (1971)

 After graduating high school in 1957, like many middle-class Iranian youths of his generation, he participated in national competitions for government-sponsored bursaries to study abroad. After a nine-month period in England dedicated to studying English, he gained admission to University of California, Los Angeles (UCLA) in Los Angeles. He received his B.A. in Bacteriology in 1964 and his Ph.D. in Microbiology and immunology in 1968. This was at the beginning of Cellular Immunology with many challenging questions, such as the cellular basis of antibody production to a vast diversity of antigens, immunologic memory and immunologic tolerance. While investigating immunological memory during his Ph.D. studies under Prof. Eli Sercarz, at UCLA and following training at Stanford University in Palo Alto, California, with Prof. Boris Rotman, he developed a sensitive enzyme-based fluorescence method to quantify minute amounts of cellular receptors for antigens. This achievement gained him recognition in immunology research with a prestigious publication in Science in 1968 (Figure 2A).^2^

 After completing his Ph.D., his government-sponsored bursary ended. However, his passion for continuing research in immunology drove him to apply for a post-doctoral fellowship at Harvard Medical School, working under the guidance of Prof. Albert Hewett Coons, the inventor of immunofluorescence technology. He focused on the role of specific antigen-binding cells in the thymus which generate the primary immune response, published in Science (Figures 2B & C).^3^ During his post-doctorate fellowship, Dr. Modabber mentored one of Prof. Coons’ students (Susan Swain) who worked on antigen binding of T- and B-cells for her Ph.D. thesis which were published in the Journal of Immunology.^4,5^ During this time, as a Harvard Fellow, he attended many courses at Harvard Medical School, although he did not get an MD. With his training, he was later employed at the WHO as a Medical Officer. In addition, he continued to work on tears as the carrier of antibodies with Prof. Sercarz and Prof. Alfred T. Sapse of UCLA. One interesting finding was that antibodies are cytophilic for corneal cells and can compete with IgE, thereby preventing allergic reactions in the eye.^6-8^ This concept was later used to develop eye-drops containing normal globulin as a relief from allergy – now replaced by synthetic compounds. After over two years of fellowship, he was offered positions, first as a research fellow and later as an Assistant Professor at Harvard School of Public Health (HSPH). He kept his academic relationship with HSPH in different positions until 1982.

## Teaching at Iranian Universities

 While maintaining his academic positions at HSPH in Boston, Dr. Modabber returned to Iran in 1971 as an Associate Professor at the Microbiology Department of Pahlavi University Medical School in Shiraz. After teaching for two years, he went back to Harvard to continue his lectures and research, focusing on animal models of trachoma under Dr. Roger Nichols, the Head of the Microbiology Department.^9,10^ Concurrently, he was also a guest lecturer of immunology at Brown University in Rhode Island, USA.

**Figure 3:**
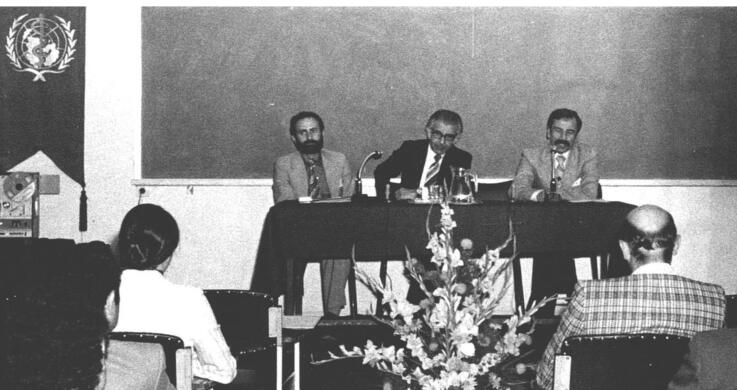
Inauguration of the Nafisi Building at TUSPH (circa 1973). Dr. Modabber (Left) with the late Dr. Shamsoddin Mofidi (Vice Chancellor of Research of Tehran University; Middle) and Dr. Abolhassan Nadim (Dean of the School of Public Health; Right)

 In 1973, Tehran University offered Dr. Modabber an Associate Professor ship position in the Department of Epidemiology and Pathobiology (later Dept. of Pathobiology) at Tehran University School of Public Health (TUSPH) where he soon became the head of the department. Nearly contemporaneously, in his new position, Dr. Modabber named a new building erected through the efforts of the late Dr. Kiarash Nafisi (who had tragically died in a car accident) after Dr. Nafisi (Figure 3).

 Moreover, Dr. Modabber established a joint program of Master’s degree with HSPH and other prestigious universities such as London and UCLA. After successful completion of the program, the students were eligible to enter a Ph.D. program at these universities. Many top Iranian immunologists, namely, the late Dr. Mohammad Faraz Nasseri (NIH, USA), the Late Dr. Abbas Hafizi, and Dr. Scheherazade Sadegh-Nasseri (Professor of Immunology at Johns Hopkins University) were among the graduates of this program. Dr. Sadegh-Nasseri, who had attended immunology courses taught by Dr. Modabber at Pahlavi University, recalls those days as follows. “His teaching style and the subject matter were fascinating, so much that inspired me to leave medical school and apply to the new Master’s program in immunology which I was accepted. I believe that had I not met Farrokh in medical school, I would have been a lost soul. I owe him my career and the love of science and immunology (personal communication, Scheherazade Sadegh-Nasseri, Johns Hopkins University, USA).

 The Pathobiology Department achieved several milestones by establishing Iran’s first HLA-typing laboratory to determine the connection between genetic background and esophageal cancer in Northern Iran,^11^ initiating immunology and genetics research on leprosy,^12^ as well as importing and breeding genetically-pure mice such as BALB/c for cutaneous leishmaniasis (CL) research. These mice were later given to Razi Institute and Pasteur Institute of Iran (PII) to support other medical research institutions. During M. Nasseri’s M.Sc. project, BALB/c mice were found highly susceptible to *Leishmania tropica major* (now known as *L. major*),^13^ establishing them as a key model for studying leishmaniases globally. Later, with his colleagues in Paris, he showed that while the initial lesion was healed, live parasites persisted in the lymphatic organs of resistant mice for extended periods.^14,15^ As a clear demonstration of premunition in leishmaniasis, Dr. Sima Rafati was able to identify the expression of live *Leishmania* in/around the scar of Dr. Modabber approximately 70 years after his recovery from CL (personal communications).

**Figure 4:**
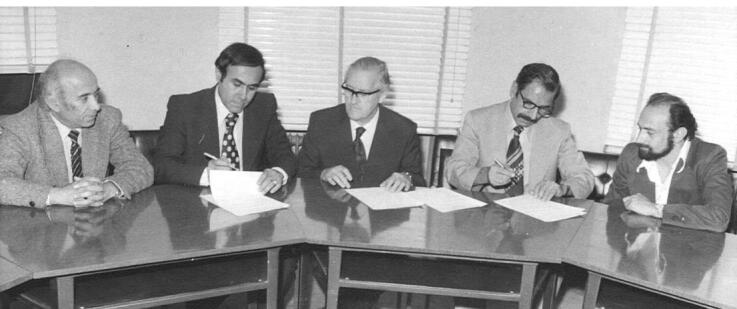
An agreement was signed between the Institute of Ophthalmology (represented by Dr. Barrie R. Jones; Center) and the School of Public Health of Tehran University. Dr. Modabber (Right) is seated close to Dr. Abolhassan Nadim (1977)

 In 1977, TUSPH signed an agreement with the prestigious Institute of Ophthalmology in London for collaboration on trachoma research in Iran (Figure 4). Dr. Modabber and Dr. Roger Nichols, former Harvard collaborators, surveyed trachoma-affected areas in Khuzestan and Fars provinces to identify treatment trial sites with known antibiotics. They found no high endemicity foci in villages between Dezful and Shiraz.^16^ Dr. Modabber reminisces that under his supervision, the Pathobiology Department was considered the Regional Immunology Training Center for the WHO Eastern Mediterranean Region Office (EMRO) in 1978. However, the final agreement was not signed as a consequence of the 1979 Iranian revolution.^1^

## General Director of the Pasteur Institute of Iran

 In 1977, an agreement signed between the Institut Pasteur in Paris (IPP) and the government of Iran caused several complaints to be leveled at the Board of Trustees of the Iranian institute. According to this agreement, the PII hosted eleven members of IPP with wages similar to what they used to receive in France which had caused a financial burden for the PII and was also a cause of resentment among the Iranian staff. Among these eleven employees, seven were not even scientists and had administrative positions while any administrative decision was beyond their jurisdictions according to Iranian law. Hence Dr. Modabber (who was still at the time with Tehran University) along with Dr. Bijan Jahangiri and Dr. Abbas Sanaati were assigned to investigate this issue. After investigating the facts, the group concluded that the agreement should be abolished. Consequently, to conclude this problem and terminate the agreement, the government offered the PII directorship to Dr. Modabber who negotiated with IPP and terminated the agreement amicably. Later, the two institutions maintained their collaboration for many years until the present time.^1^

 Following the completion of this mission, Dr. Modabber offered his resignation as the General Director of the PII. Due to positive moves which had initiated during his short tenure, such as improving the well-being, salaries, and housing conditions of the employees along with strategic planning for the development of the institute, such as the initiation of a production complex, his resignation was not accepted. At the beginning of 1979, he joined the sit-in of Tehran University faculty in support of the demand for political and academic independence of universities^1^. The new transitional government subsequently appointed a new Director of PII, and he returned to Tehran University after its reopening. However, the situation in his department had changed drastically due to the departure of several faculty members and his foreign student. Faced with the uncertainties surrounding educational policies at the time, he decided to request a sabbatical leave after a few months. Since his request for a sabbatical leave was denied (as the university had discontinued the sabbatical program), he took an unpaid leave to pursue his research at IPP.^1^

## Research on Leishmaniases

 With a moderate research grant from IPP and the WHO, Dr. Modabber joined Dr. Louis Selim Chedid’s laboratory at IPP and started intense research on CL.^15,17^ They concluded that CL is a local skin lesion but the infection is systemic in mice of different genetic backgrounds, persisting for years post-healing, providing immunity to reinfection. Having had CL in childhood may explain why he never got leishmaniasis during his long career despite working in endemic areas like Sudan, India, Bangladesh, and South America. Their collaboration continued on immunology topics, including muramyl dipeptide as adjuvants till 1982.^14,18-24^

 After returning to the US, Dr. Modabber held positions as Head of the Immunology Departments at Tulsa University (Oklahoma) and Syntex Institute of Biology in Palo Alto (California). Aside from providing an ideal environment to further pursue his leishmaniases research as a model of inflammation,^25^ the latter move allowed him to be closer to his sons, Zia and Ramin who were studying at UC Berkeley, as well as his nephew and adopted son, Nader. In 1983, Dr. Modabber was invited to review the Leishmaniasis Division at the WHO’s Tropical Disease Research (TDR) in Geneva. Shortly, he was offered a position at TDR, fulfilling his dream of engaging with various countries working on leishmaniases, especially Iran. Initially a Medical Officer, he later became a Scientist on *Leishmania* research, including immunology, chemotherapy, and epidemiology and eventually, the Coordinator of Research Capability Strengthening, a significant position at TDR.

 He advocated for a vaccine as the most effective, logical, and affordable tool to control leishmaniases. Initially, he proposed a first-generation *Leishmania* vaccine, for lack of a better candidate, composed of whole-killed parasites, based on previous research done in Venezuela and Brazil.^26^ A leishmanization program, led by Prof. Nadim in Iran, involved intradermal inoculation of live *L. major* to induce protective immunity in soldiers during the Iran-Iraq war.^27^ This method later served as a live challenge, requiring proper production and storage for human use. With grants from TDR, Dr. Reza Hashemi-Fesharaki at Razi Vaccine and Serum Research Institute established the production of a killed vaccine candidate under WHO guidance and supervision. Subsequently, live parasites were generated for use as a challenge to assess experimental vaccines. Dr. Ali Khamesipour and colleagues demonstrated the repeatability of the live *Leishmania* challenge.^28^ Live challenge, used successfully for other diseases like malaria, is a cost-effective way to evaluate candidate vaccines compared to field trials.

 After the approval of the procedure by the WHO, Phase-1 safety and dose-finding studies began using killed parasites and the PII BCG vaccine as an adjuvant^29^ under Dr. Yahya Dowlati’s guidance at Center for Research and Training in Skin Diseases and Leprosy (CRTSDL) of Tehran University. However, by the time the vaccine was produced under GMP-like conditions, the war and the leishmanization program had ended. To assess vaccine efficacy, TDR-funded studies were carried out in CL-endemic zones near Isfahan and Bam cities in Iran. The trial outside Isfahan, directed by Dr. Ali Z. Momeni,^30^ and the trial in Bam, directed by Dr. Iraj Sharifi, both published in *The Lancet*,^31^ offered a glimpse of hope as they showed that a fraction of the population had converted their skin tests to positive after vaccination, indicating protection against CL. Guided by Good Clinical Practices (GCP), Iran established its first ethical committee for these human studies with the support of the late Dr. Bijan Sadrizadeh^32^ and workshops funded by TDR were conducted to meet the GCP requirements. While the trials yielded interesting findings, the vaccine’s efficacy was deemed unsatisfactory.^30,33^ Subsequently, Razi Institute developed alum-precipitated killed *L. major* plus BCG, demonstrating higher immunogenicity and significant efficacy in Sudan both as an adjunct to chemotherapy for the treatment of chronic PKDL^34,35^ and as a prophylactic vaccine against VL. Unfortunately, it was never properly tested in Iran using the live challenge, after Dr. Modabber departed from TDR. He believes that until a safe efficacious and affordable vaccine for leishmaniasis is developed, the Alum-ALM + BCG is the best alternative and should be tested properly.

 Under his direction, TDR assisted Dr. Mohammad-Hossein Alimohammadian to launch a semi-industrial GMP-like facility at PII for producing Leishmanin, a reagent composed of killed *Leishmania* promastigotes. The reagent is used for a skin test that detects DTH to leishmanial antigens. The test is crucial for epidemiological assessments and vaccine response evaluations and remains positive in cured patients who are generally immune to reinfection. The potent Leishmanin from PII was later tested in many countries. It was selected and distributed by TDR, to become a global standard reference antigen for leishmaniases studies.^36^ Dr. Mohebali and colleagues demonstrated its relative effectiveness as a vaccine in protecting dogs.^37^ Unfortunately, these efforts were disrupted after Dr. Modabber retired from the WHO and the concept of vaccinating volunteers with Leishmanin or other candidate vaccines, followed by leishmanization as a live challenge, was never followed properly. During Dr. Modabber’s 16-year tenure at TDR/WHO, over 30 grants supported training, vaccine, and treatment studies in Iran. These included training and a small construction (one grant to Dr. Mahboudi for the construction of a GMP-like facility in the PII). Beneficiary institutions included PII, TUSPH, CRTSDL, Razi Institute, Isfahan University (for vaccine trials with a locally made topical ointment),^38^ and Kerman University.

 Collaborations with Iranian and international scientists greatly influenced *Leishmania *studies in Iran. Visitors during this time fondly recall the impactful field activities. For instance, Dr. David Sacks mentions, “I had the remarkable opportunity of visiting Iran with Farrokh on many occasions during the 1990s in connection with activities sponsored by TDR. As Secretary of the Leishmaniasis Steering Committees of TDR, Farrokh invited me to chair the immunology sub-committee, which is where I experienced firsthand the passion, energy and scientific excellence that he brought to the shaping of research priorities aimed at the development of new tools to combat Leishmaniasis. Farrokh was a unifying force behind this global effort. It was under his guidance that TDR supported a series of clinical trials of so-called ‘first generation’ vaccines against Leishmaniasis. These were major activities in different parts of the world that required his constant input and expertise, starting with discussions surrounding the scientific rationale for a whole-cell killed vaccine, the use of BCG as an adjuvant, the production of a vaccine under GMP standards, and the proper design and conduct of safety and efficacy trials. He always emphasized the vital role that TDR plays in training and mentoring. The number of scientists around the world who benefitted from TDR support and who remain in Leishmaniasis research or related fields even today is one of the lasting legacies of the program during his leadership.” (personal communication, David Sacks, National Institutes of Health, USA).

**Figure 5:**
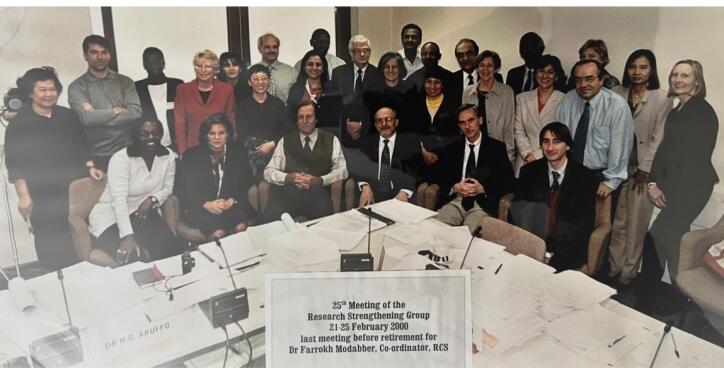
The last Research-Strengthening Group was coordinated by Dr. Modabber (the 3^rd^ seated person from Right) at TDR, 2000

 Dr. Moulton remembers, “It was my good fortune to work with him during several trips to Iran during 1997‒2000. I can only use the word ‘thrilling’ to describe seeing Dr. Modabber fully in his element, exhibiting a combination of near-childish excitement, scientific integrity, and tact in a variety of situations. His ability to focus simultaneously on the critical details of the project, while not losing sight of the big picture, was as inspiring as it was impressive.” (personal communication, Lawrence H. Moulton, Johns Hopkins University, USA). Other major collaborators with Dr. Modabber in Iran studies were A. Mitchison, P.G. Smith, J. Cerney, K. Nasseri, S. Mofidi, A. Nadim, R. Nategh, N. Mohagheghpour, S. Noazin, G. Rook, J. Stanford, M. Takasugi, R. Nichols and M. Essex. After mandatory retirement at sixty, Dr. Modabber had to leave TDR at the turn of the century (Figure 5).

## IDRI, DNDi, and Later Engagements

 Dr. Modabber directed the Infectious Disease Research Institute (IDRI) in Seattle for four years upon returning to the US. IDRI, a non-profit organization established in 1994, focuses on developing vaccines and therapeutics for malaria and VL with support from TDR and the Gates Foundation. In Sudan, he facilitated the development of a second-generation defined Leishmania vaccine.^39^ In 2004, he joined the Drugs for Neglected Diseases initiative (DNDi) in Geneva, serving in various roles and lastly, as a Senior Advisor. At DNDi, he oversaw a pivotal Phase-3 trial, combining previously-used drugs for VL treatment (*i.e*. low-doses of Ambisome plus other drugs such as Paromomycin, Antimonial, and Meltefosin), resulting in definitive outcomes in India and Bangladesh.^40^ These combinational therapies not only shortened treatment and reduced side effects but also cut overall costs, addressing key concerns for diseases in impoverished areas.

**Figure 6:**
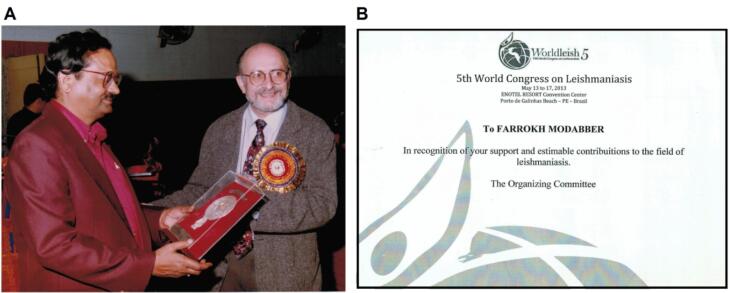
** (A)** Dr. Modabber accepts an achievement award from the Institute of Medical Sciences (Banaras Hindu University, Varanasi, India) for research-strengthening and collaboration with the University (1999).** (B)** Citation conferred to Dr. Modabber during the 5^th^ World Congress on Leishmaniasis for recognition of his support and contribution to the field of Leishmaniasis (2013)

 Dr. Modabber is recognized for his dedication to advancing leishmaniasis research, such as receiving an award for Teaching Good Clinical Practices and Research-Strengthening Activities at a TDR meeting in India in 1999 (Figure 6A) and the prestigious “Dr. Rajendra Prasad Memorial Oration Award” from Rajendra Memorial Research Institute of Medical Sciences (RMRIMS) in Patna, India, as recognition of his Research-Strengthening activities and Achievements for Therapy of VL in India in 2010. Additionally, he was honored at the WorldLeish 5 Congress in Brazil in 2013 for his contributions to the field (Figure 6B).

 Dr. Modabber emphasizes that while reaching the goal is the ultimate achievement and joy, each step that brings us closer to the goal is an achievement. He maintains that his life was not in vain, because after decades of relentless efforts, there is still not a good treatment or an efficacious and affordable vaccine for CL. Meanwhile, for each properly designed and executed experiment/trial, some valuable knowledge is gained. For instance, his work on combined chemotherapy in India has improved VL patient care, benefiting neglected populations. Progress in research quality in Iran has led to recognition for Iranian scientists in the global Leishmaniasis community. On the top of his list, Dr. Sima Rafati stands out for her tireless efforts and dedication in securing grants, establishing collaborations, and mentoring top-tier graduate students at the PII. Dr. Modabber is proud of his contributions to Iranian scientists and believes that with more support and independence, they will excel in tackling the challenges of this neglected global disease.

 Dr. Modabber was married three times, to Lillian Guttman-Roth in 1961, the late Minou Bayat-Modabber in 1969, and to Marlies Haegglund, an Austrian old colleague at the WHO in 2019. He is proud of his offspring: sons, Zia, a prominent Lawyer in Los Angeles, Ramin, a prominent orthopedic surgeon and mentor in Santa Monica, and Nader, an achieved artist, in Santa Monica; his daughter, Yalda, Founder and now Executive Director of Golestan School (http://www.golestankids.com/), in Berkeley, California. Dr. Modabber is presently retired and offers consultation to DNDi regarding the leishmaniasis projects and reviews proposals on vaccines for some granting agencies when required. He lives partially in California, Austria and Iran.

 In closing, it is worth noting Dr. Rook’s assertion: “I have the impression that Farrokh spent much of his career advising, organizing and helping others to achieve success while encouraging and assisting the WHO programs for tropical diseases. Farrokh Modabber is an inspirational thinker and advisor, and an utterly charming man full of culture, humor and kindness in addition to his scientific acumen.” (personal communication, Graham Rook, University College London, UK).

## Conclusion

 Dr. Farrokh Modabber has been an advocate for scientific development in Iran and beyond. His remarkable journey serves as an inspiration to future generations of scientists, demonstrating that a strong foundation of quality education and unwavering commitment can drive transformative change in global health.

## References

[1] Hosseini F, Nikbin B, Jaffari R. History of Immunology and Allergy in Iran and the World (Chapter 10). Tehran: Ebn-e-Sina Press; 2018. p. 273-86. [Persian].

[2] Sercarz EE, Modabber F (1968). Antigen binding to cells: determination by enzymic fluorogenic group hydrolysis. Science.

[3] Modabber F, Morikawa S, Coons AH (1970). Antigen-binding cells in normal mouse thymus. Science.

[4] Swain S, Modabber F, Coons AH (1976). Characterization of T and B antigen-binding cells for beta-galactosidase I beta-galactosidase-binding cells in the thymus and spleen of normal mice. J Immunol.

[5] Swain S, Modabber F, Coons AH (1976). Characterization of T and B antigen-binding cells for beta-galactosidase II T antigen-binding cells. J Immunol.

[6] Modabber F, Sapse AT (1971). Competition of normal gamma-globulins with cytophilic antibody for corneal cells. Int Arch Allergy Appl Immunol.

[7] Modabber F, Sapse AT, Sercarz EE (1969). Tears as carriers of antibodies II. Ann Ophthalmol.

[8] Modabber F, Sapse AT, Sercarz EE (1969). Presence of antibodies cytophilic to corneal epithelium in rabbit tears. Invest Ophthalmol.

[9] Watson RR, MacDonald AB, Murray ES, Modabber F (1973). Immunity to chlamydial infections of the eye 3 Presence and duration of delayed hypersensitivity to guinea pig inclusion conjuctivitis. J Immunol.

[10] Modabber F, Bear SE, Cerny J (1976). The effect of cyclophosphamide on the recovery from a local chlamydial infection Guinea-pig inclusion conjunctivitis (GPIC). Immunology.

[11] Hashemi S, Dowlatshahi K, Day NE, Kmet J, Takasugi M, Mohaghpour N (1979). Esophageal cancer studies in the Caspian Littoral of Iran: introductive assessment of the HLA profile in patients and controls. Tissue Antigens.

[12] Mohagheghpour N, Tabatabai H, Mohammad K, Ramanujam K, Modabber F (1979). Histocompatibility antigens in patients with leprosy from Azarbaijan, Iran. Int J Lepr Other Mycobact Dis.

[13] Nasseri M, Modabber F (1979). Generalized infection and lack of delayed hypersensitivity in BALB/c mice infected with Leishmania tropica major. Infect Immun.

[14] Leclerc C, Modabber F, Deriaud E, Djoko-Tamnou J, Chedid L (1982). Visceral Leishmania tropica infection of BALB/c mice: cellular analysis of in vitro unresponsiveness to sheep erythrocytes. Infect Immun.

[15] Djoko-Tamnou J, Leclerc C, Modabber F, Chedid L (1981). Studies on visceral Leishmania tropica infection in BALB/c mice I Clinical features and cellular changes. Clin Exp Immunol.

[16] Modabber F, McComb D, Pourmand MH, Nichols R (1978). Preliminary survey of trachoma in 11 selected villages in Khouzestan and Fars. Iran J Public Health.

[17] Leclerc C, Modabber F, Deriaud E, Cheddid L (1981). Systemic infection of Leishmania tropica (major) in various strains of mice. Trans R Soc Trop Med Hyg.

[18] Colle JH, Truffa-Bachi P, Chedid L, Modabber F (1983). Lack of general immunosuppression during visceral Leishmania tropica infection in BALB/c mice: augmented antibody response to thymus-independent antigens and polyclonal activation. J Immunol.

[19] Bahr GM, Modabber F, Rook GA, Mehrotra ML, Stanford JL, Chedid L (1982). Absence of antibodies to muramyl dipeptide in patients with tuberculosis or leprosy. Clin Exp Immunol.

[20] Bahr GM, Carelli C, Audibert F, Modabber F, Chedid L (1982). Analysis of the antigenic relationship of various derivatives of n-acetyl-muramyl-l-ala-d-isoglutamine (MDP), using anti-MDP antibodies. Mol Immunol.

[21] Bahr GM, Eshhar Z, Ben-Yitzhak R, Modabber F, Arnon R, Sela M (1983). Monoclonal antibodies to the synthetic adjuvant muramyl dipeptide: characterization of the specificity. Mol Immunol.

[22] Phillips NC, Bahr GM, Modabber F, Chedid L (1984). Modulation of the growth of murine thymoma cell lines having different Lyt-phenotypes by MDP and MDP(D-D): macrophage-mediated inhibition of in vitro cell growth. Int J Immunopharmacol.

[23] Bahr GM, Modabber F, Morin A, Terrier M, Eyquem A, Chedid L (1984). Regulation by muramyl dipeptide (MDP) of the lymphoproliferative responses and polyclonal activation of human peripheral blood mononuclear cells. Clin Exp Immunol.

[24] Scott MT, Bahr GM, Moddaber F, Afchain D, Chedid L (1984). Adjuvant requirements for protective immunization of mice using a Trypanosoma cruzi 90K cell surface glycoprotein. Int Arch Allergy Appl Immunol.

[25] Mirkovich AM, Galelli A, Allison AC, Modabber F (1986). Increased myelopoiesis during Leishmania major infection in mice: generation of ‘safe targets’, a possible way to evade the effector immune mechanism. Clin Exp Immunol.

[26] Noazin S, Modabber F, Khamesipour A, Smith PG, Moulton LH, Nasseri K (2008). First generation leishmaniasis vaccines: a review of field efficacy trials. Vaccine.

[27] Mostafavi E, Haghdoost A, Yavari P, Chaman R, Mesdaghinia A, Enayatrad M. Dr. Abolhassan Nadim, founder of modern epidemiology in Iran. Iran J Epidemiol 2018;13(4):264-72. [Persian].

[28] Khamesipour A, Dowlati Y, Asilian A, Hashemi-Fesharki R, Javadi A, Noazin S (2005). Leishmanization: use of an old method for evaluation of candidate vaccines against leishmaniasis. Vaccine.

[29] Bahar K, Dowlati Y, Shidani B, Alimohammadian MH, Khamesipour A, Ehsasi S (1996). Comparative safety and immunogenicity trial of two killed Leishmania major vaccines with or without BCG in human volunteers. Clin Dermatol.

[30] Momeni AZ, Jalayer T, Emamjomeh M, Khamesipour A, Zicker F, Labaf Ghassemi R (1999). A randomised, double-blind, controlled trial of a killed L major vaccine plus BCG against zoonotic cutaneous leishmaniasis in Iran. Vaccine.

[31] Sharifi I, FeKri AR, Aflatonian MR, Khamesipour A, Nadim A, Mousavi MR (1998). Randomised vaccine trial of single dose of killed Leishmania major plus BCG against anthroponotic cutaneous leishmaniasis in Bam, Iran. Lancet.

[32] Bardestani F, Marandi SA, Malekzadeh R, Nadim A, Malekafzali H, Bagheri Lankarani K (2023). In commemoration of Dr Bijan Sadrizadeh, a prominent physician and expert in the field of public health in Iran and around the world. Arch Iran Med.

[33] Alimohammadian MH, Khamesipour A, Darabi H, Firooz A, Malekzadeh S, Bahonar A (2002). The role of BCG in human immune responses induced by multiple injections of autoclaved Leishmania major as a candidate vaccine against leishmaniasis. Vaccine.

[34] Khalil EA, Musa AM, Modabber F, El-Hassan AM (2006). Safety and immunogenicity of a candidate vaccine for visceral leishmaniasis (alum-precipitated autoclaved Leishmania major + BCG) in children: an extended phase II study. Ann Trop Paediatr.

[35] Musa AM, Khalil EA, Mahgoub FA, Elgawi SH, Modabber F, Elkadaru AE (2008). Immunochemotherapy of persistent post-kala-azar dermal leishmaniasis: a novel approach to treatment. Trans R Soc Trop Med Hyg.

[36] Alimohammadian MH, Kivanjah M, Pak F, Gaznavia A, Kharazmi A (1993). Evaluation of the efficacy of Iran leishmanin and comparison with leishmanins from Wellcome (UK) and Roma (Italy) in cured cutaneous leishmaniasis patients. Trans R Soc Trop Med Hyg.

[37] Mohebali M, Khamesipour A, Mobedi I, Zarei Z, Hashemi-Fesharki R (2004). Double-blind randomized efficacy field trial of alum precipitated autoclaved Leishmania major vaccine mixed with BCG against canine visceral leishmaniasis in Meshkin-Shahr district, IR Iran. Vaccine.

[38] Asilian A, Jalayer T, Nilforooshzadeh M, Labaf Ghassemi R, Peto R, Wayling S (2003). Treatment of cutaneous leishmaniasis with aminosidine (paromomycin) ointment: double-blind, randomized trial in the Islamic Republic of Iran. Bull World Health Organ.

[39] Coler RN, Skeiky YA, Bernards K, Greeson K, Carter D, Cornellison CD (2002). Immunization with a polyprotein vaccine consisting of the T-cell antigens thiol-specific antioxidant, Leishmania major stress-inducible protein 1, and Leishmania elongation initiation factor protects against leishmaniasis. Infect Immun.

[40] Sundar S, Sinha PK, Rai M, Verma DK, Nawin K, Alam S (2011). Comparison of short-course multidrug treatment with standard therapy for visceral leishmaniasis in India: an open-label, non-inferiority, randomised controlled trial. Lancet.

